# Association Between Chrononutrition Patterns and Multidimensional Sleep Health

**DOI:** 10.3390/nu16213724

**Published:** 2024-10-31

**Authors:** Namhyun Kim, Rachel Kolko Conlon, Samaneh Farsijani, Marquis Samuel Hawkins

**Affiliations:** 1 Department of Epidemiology, University of Pittsburgh, Pittsburgh, PA 15261, USA; nak264@pitt.edu (N.K.); samaneh.farsijani@pitt.edu (S.F.); 2 Department of Psychiatry, University of Pittsburgh, Pittsburgh, PA 15261, USA; kolkorp@pitt.edu; 3 Department of Psychology, University of Pittsburgh, Pittsburgh, PA 15261, USA

**Keywords:** sleep, eating, circadian rhythm, meal timing, chrononutrition

## Abstract

Background/Objectives: Sleep health has been associated with diet quality, but the relationship between chrononutrition patterns and multidimensional sleep health is unclear. This study identifies chrononutrition patterns among U.S. adults and examines their associations with multidimensional sleep health. Methods: This cross-sectional analysis used data from the 2017–2020 National Health and Nutrition Examination Survey. Chrononutrition behaviors were assessed using two 24 h dietary recalls. Latent profile analysis was used to identify chrononutrition profiles. Multivariable survey regression models determined the associations between chrononutrition patterns and sleep health dimensions. Results: The sample included 5228 subjects with a median age of 49 years. Of the sample, 52% of the participants were female, and 65% were White. In adjusted models, each additional hour between wake time and first instance of eating was associated with a 19% increase in the odds of poor timing (sleep midpoint < 2:00 a.m. or >4:00 a.m.; 95% CI: 1.07–1.33) and a 21% increase in poor duration (<7 or >9 h/night; 95% CI: 1.09–1.33). Each additional hour between last eating and bedtime was associated with 9% higher odds of poor duration (95% CI: 1.03–1.16). A one-hour longer eating window was associated with 10% lower odds of poor timing (95% CI: 0.84–0.98). We identified five chrononutrition profiles: Typical Eating (reference), Early Finished Eating, Later Heavy Eating, Extended Window Eating, and Restricted Window Eating. The Later Heavy Eating profile exhibited 96% higher odds of poor timing (95% CI: 1.09–3.51) and the Restricted Window Eating profile had 94% higher odds of poor duration (95% CI: 1.10–3.43). Conclusions: These findings highlight the importance of unique chrononutrition patterns in relation to multidimensional sleep health. We provide a framework for future studies to identify personalized chrononutrition interventions and their role in improving sleep health.

## 1. Introduction

Irregular sleep, poor sleep quality, daytime sleepiness, and short and long sleep durations are associated with increased all-cause and cardiovascular mortality and a range of cardiometabolic (e.g., obesity and hypertension) and cognitive conditions (e.g., depression and dementia) [1,2,3,4,5,6,7,8]. The American Heart Association (AHA) recently recognized the significance of sleep health by adding it to its ‘Life’s Essential 8’ (formerly Life’s Simple 7) cardiovascular health metric [9]. However, a significant proportion of US adults fail to meet the AHA, American Academy of Sleep Medicine, or National Sleep Foundation sleep health recommendations [4,10,11]. Therefore, research that seeks to identify the factors contributing to poor sleep health is crucial.

Diet has been identified as playing a significant role in improving sleep health [12]. A healthy diet quality that includes fruits and vegetables is associated with better sleep quality and duration [13,14,15,16]. However, the impact of the timing of dietary intake on sleep remains largely unexplored. Emerging evidence suggests the timing and frequency of food intake (i.e., chrononutrition) can influence health by entraining the body’s circadian clock [17]. Chrononutrition encompasses not only the types of foods consumed but also the timing, frequency, and distribution of food intake throughout the day, all of which can significantly impact metabolic processes and overall health [18,19,20].

However, there is a pressing need to determine how the timing of intake influences sleep health. Current evidence from small-scale randomized controlled trials (n < 100) is inconsistent, and the generalizability of the findings is questionable due to their limited scope (e.g., university students or patients with sleep apnea) [21,22,23]. Moreover, the extant literature is limited due to the focus on single chrononutrition behavior (e.g., breakfast skipping, eating windows) and the sleep health domain (typically quality or duration) [24,25,26,27]. Chrononutrition behaviors are interrelated, likely creating a synergy that impacts multidimensional sleep health [28]. However, few studies have examined the relationship between chrononutrition patterns and multidimensional sleep health [29,30,31]. Understanding the complex relationship between chrononutrition patterns and multidimensional sleep health could better inform policy and health promotion efforts.

This descriptive study aims to (1) employ latent profile analysis to identify distinct chrononutrition profiles among U.S. adults and (2) examine the associations between chrononutrition patterns and sleep health domains individually and within a multidimensional sleep health framework, utilizing nationally representative data from the National Health and Nutrition Examination Survey (NHANES). We anticipate that we will observe that consuming the first meal far from wake time, consuming the last meal close to bedtime, consuming most calories later in the day, and having a longer eating window will be associated with poor sleep health.

## 2. Materials and Methods

### 2.1. Study Design

This is a cross-sectional analysis of the National Health and Nutrition Examination Survey (NHANES), a national surveillance survey designed to assess the health and nutrition status of noninstitutionalized children and adults. NHANES collects data on a nationally representative sample of approximately 10,000 individuals each two-year cycle by over-sampling individuals aged 60 and above, and who identify as African American and/or Hispanic, to produce reliable statistics [32]. Due to the COVID-19 pandemic, the 2019–2020 survey cycle was incomplete and thus not representative. Therefore, the partial 2019–March 2020 data were combined with the complete 2017–2018 dataset to form a nationally representative 2017–March 2020 pre-pandemic dataset. This incorporated data from primary sampling units chosen through two distinct sampling designs: the 2017–2018 data following the 2015–2018 design and the 2019-March 2020 data based on the 2019–2022 design [32].

The survey includes comprehensive in-home health interviews and detailed physical examinations conducted in mobile examination centers [33]. The NHANES study protocols were approved by the National Center for Health Statistics Ethics Review Board (Protocol #2018-01), and all participants provided written informed consent before participating. For this study, we used the 2017 to 2020 pre-pandemic cycles of the NHANES, the most recent and available dataset (N = 15,560). The inclusion criteria were adults aged 18 and over who had completed two 24 h diet recall and sleep questionnaires. Exclusion criteria included pregnancy, BMI < 14 or >56 kg/m^2^, and extremely high- or low-calorie intake (i.e., >6500 or <600 calories). For the present study, 10,332 participants were excluded, and the final sample size was 5228 (Figure S1).

### 2.2. Diet Assessment

The dietary data collection involved the mobile examination center (MEC) in-person interview, which included a 24 h dietary recall, information on supplement use, and a series of post-recall questions, using three-dimensional and two-dimensional measuring guides to help participants accurately estimate portion sizes. A phone follow-up interview (PFU) was conducted 3–10 days after the first recall to collect a second 24 h dietary recall [34]. One weekend and one weekday were included in the two dietary recalls, representing participants’ dietary habits [34].

Eating episodes were defined as consuming a meal, snack, or beverage containing calories (>0 kcal). In the present study, we averaged the data from Day 1 and Day 2 to assess the timing of the first and last eating episodes, hours between waketime and the first meal, hours between the last meal and bedtime, hours between the awake midpoint and 50% caloric intake timing, and the duration of the eating window. Considering NHANES’s 24 h recall standard based on midnight, the first eating time was defined as the initial caloric consumption after 12:00 a.m., and the last eating time was the final caloric consumption before the next 12:00 a.m. We calculated the eating window as the duration between first and last intake.

### 2.3. Sleep Health Assessment

Sleep health was assessed by self-report questionnaires. Participants reported their typical sleep and wake times during weekdays/workdays and weekends, any sleep issues reported by a doctor, and their frequency of feeling overly sleepy during the day over the past month. *Sleep regularity* was quantified as the variance in sleep midpoint between weekdays and weekends, and then categorized into two levels (Good: ≤2 h and Poor: >2 h) [11,28]. *Sleep satisfaction* was indirectly measured with doctor-reported sleep troubles (Good: not reporting sleep problems, and Poor: reporting sleep problems to a doctor) [11,35,36,37,38,39]. *Alertness* was determined based on the frequency of daytime sleepiness reported. Across the five levels of frequency for sleepiness, designed as never, rarely (once a month), sometimes (2–4 times/month), often (5–15 times/month), and almost always (16–30 times/month), we consolidated into two levels for analysis: (1) Good: never, rarely, and sometimes, and (2) Poor: often and almost always [11]. *Sleep timing* was determined using the midpoint between going to bed and waking up, with an adjusted average for both weekdays and weekends. Sleep timing was then categorized into 2 levels (Good: between 2:00 a.m. and 4:00 a.m., and Poor: before or after 2:00 a.m. and 4:00 a.m.) [11,28]. *Sleep duration* was calculated by subtracting the reported bedtime from the waketime, with separate reports for weekdays and weekends. The overall sleep duration was then derived as a weighted mean of these weekday and weekend durations and then categorized (Good: from 7 to 9 h, Poor: less than 7 h or more than 9 h) [40]. A *multidimensional sleep health composite score* was calculated by totaling the number of good indicators across various sleep domains, ranging from 0 to 5, where a higher score signified better sleep health.

### 2.4. Covariates and Descriptive Characteristics

Demographic characteristics were collected through questionnaires by trained interviewers in participants’ homes. The demographic variables included age, gender, race, employment status, income, and education level. Depression status was assessed using the Patient Health Questionnaire (PHQ-9), with trained interviewers assessing the frequency of depression symptoms. Individuals with a score of ≥15 were classified as having depression, whereas those with scores < 15 were categorized as not having depression [41]. Total caffeine intake amount was calculated based on the consumption of food and beverages, with the average of Day 1 and Day 2. Smoking status was classified based on serum cotinine concentration, with levels > 10 ng/mL indicating current smoking and levels below this threshold indicating non-smoking [42]. For alcohol consumption, participants indicating they had never consumed alcohol were classified as ‘no’. Those who had consumed alcohol answered a follow-up question about average weekly intake over the past 12 months, leading to classifications of ‘moderate’ (men: ≤2, women: ≤1 drinks/day), or ‘heavy’ drinking (men: >2, women: >1 drinks/day) [43]. Participants self-reported using supplements for relaxation, stress, or sleep on either Day 1 or Day 2.

Physical activity levels were assessed using the Global Physical Activity Questionnaire (GPAQ). Participants who reported engaging in vigorous or moderate-intensity sports, fitness, or recreational activities in a typical week were asked to detail the duration and frequency of these activities. Adherence to guidelines was based on achieving ≥150 min of moderate or 75 min of vigorous activity weekly, or an equivalent combination [44]. The healthy eating index 2015 (HEI-2015) was calculated based on individual consumed foods and beverages (range: 0–100, with higher scores indicating better dietary quality). Participants also self-reported whether they were following a specific diet for weight loss or other health-related reasons (i.e., diets focused on weight loss or low-calorie intake, low fat or cholesterol, low salt, sugar-free or low sugar, low or high fiber, and diabetic diets), which we categorized as yes/no.

### 2.5. Statistical Analysis

Given the complex sampling design of NHANES, all analyses utilized sample weights, clustering, and stratification to ensure representativeness of the U.S. adult population [32]. We excluded participants if there were missing data for any key exposure variables or primary outcome variables (N = 4465). We compared the characteristics of participants with complete data to those with incomplete data to identify bias (Table S1).

We used latent profile analysis (LPA) to identify distinct chrononutrition profiles. LPA identifies unmeasured latent classes (i.e., chrononutrition profiles) using continuous measured indicators [45]. To ascertain the optimal number of classes, we fit models ranging from 2 to 10 classes. The most suitable model was selected based on statistical criteria and practical considerations, including: (1) Bayesian Information Criterion (BIC) and Akaike Information Criterion (AIC), where lower values indicate a better model fit; (2) the Likelihood Ratio Test (LRT), with a significance level set at alpha of 0.05 to assess the improvement of fit between models; (3) a minimum class size of 5% to ensure sufficient sample size in each class; and (4) the overall interpretability of the classes to ensure the model’s practical applicability. Descriptive statistics, including mean, standard deviation, and proportions, were calculated to compare demographic characteristics and covariates across the chrononutrition profiles.

Next, we conducted multivariable survey-weighted linear and logistic regression analyses to explore the associations between individual chrononutrition behaviors, the identified chrononutrition profiles, each sleep domain (Regularity, Satisfaction, Alertness, Timing, and Duration), and composite sleep health score [32]. The adjusted models included age, race, education, income, marital status, body mass index, depression, diet quality, alcohol use, caffeine, and total calories to control for potential confounding factors based on prior studies and the research team’s expertise.

We conducted sensitivity analyses to assess the robustness of our findings under various conditions. Specifically, we defined sleep regularity based on the difference in sleep duration between weekdays/workdays and weekends. Additionally, we categorized sleep duration into binary variables, with thresholds set at less than 6 h and greater than 10 h, thereby defining “Good” sleep as between 6 to 10 h and “Poor” sleep as less than 6 or more than 10 h [40]. Our sensitivity analysis also included those who were excluded due to BMI and calorie exclusion criteria. Since the primary and sensitivity analyses yielded almost identical results, we included only the primary analysis in this manuscript.

The statistical analyses were performed using R version 4.4.1. We used the survey package in R (version 4.4-2) to account for the complex survey design. The two-day dietary weights were used to account for bias [46]. We used the gtsummary package (version 2.0.3) [47] to generate descriptive and regression tables and the depmixS4 packages (version 1.5-0) [48] for latent variable model analysis. All statistical analyses adopted a significance level of 0.05.

## 3. Results

Among the 15,560 respondents in the NHANES 2017–2020 pre-pandemic cycle, 9693 were adults, and 5228 were included in the final analytic sample (Figure S1). Compared to the overall adult population, individuals included in the analysis were older (median age 49 years vs. 47 years), had a higher proportion of white individuals (65% vs. 62%), had a higher education level beyond high school (66% vs. 62%), had higher income, a higher proportion were married/partnered (64% vs. 62%), a lower proportion currently smoked (20% vs. 23%), and more drank alcohol moderately (48% vs. 46%) (Table S1).

We identified five unique chrononutrition profiles through LPA. Both AIC and BIC values decreased as the number of classes increased, but there was minimal reduction beyond five classes (Figure S2). The six-class solution identified class sizes below our a priori threshold (<5%). Therefore, considering statistical indicators, class size, and interpretability, and to avoid overfitting, we selected a five-class model.

### 3.1. Demographic and Behavioral Differences in Chrononutrition Profiles

The Extended Window Eating profile had the highest age (median 55 years), calorie intake (mean 2230 kcal), caffeine consumption (median 164 mg), and Healthy Eating Index (mean 53) values, while the Restricted Window Eating profile had the lowest (i.e., median age 41 years, median caffeine consumption 69 mg, and mean Healthy Eating Index 45). Later Heavy Eating was more common among non-Hispanic Black individuals (30%), while Restricted Window Eating was more common among those with lower education (17%). Higher income and marriage were more common in the Typical Eating profile (35% and 34%, respectively). Depression was higher in the Restricted Window Eating profile (Table 1).

### 3.2. Chrononutrition Behavior Differences in Chrononutrition Profiles

The Typical Eating profile had an initial eating episode 50 min after waking and the final eating episode 2.67 h before bedtime. This profile closely aligned with the overall population mean (e.g., eating window: 12.74 h and caloric midpoint: 7 min after waking midpoint). In the Early Finished Eating profile, the first eating episode happened 1.54 h after waking, and the final eating episode concluded 4.22 h before bedtime. The eating window lasted 10.5 h, with the caloric midpoint occurring 30 min earlier than the waking midpoint. The Later Heavy Eating profile began its first eating episode 2.83 h after waking, with the last eating episode occurring 1.69 h prior to sleep. Although the eating window was 11.80 h, similar to the Typical Eating profile, the caloric midpoint was 1.68 h later than the midpoint of the waking period. The Extended Window Eating profile initiated eating 49 min after waking, and the final eating episode took place 1.01 h before sleep, resulting in the longest eating window of 14.39 h. The Restricted Window Eating profile started eating around 3.44 h after waking, the latest among all profiles. The final eating episode was 6.16 h before bedtime, the earliest among the profiles, with the shortest eating window of 9.01 h. The caloric midpoint was 16 min later than the waking midpoint (Table 2).

### 3.3. Chrononutrition Profiles and Behaviors Related to Multidimensional Sleep Health

Compared to the Typical Eating profile, the Later Heavy Eating profile had a 0.23-point lower composite sleep health score (95% CI: −0.37, −0.1), and the Restricted Window Eating profile had a 0.55-point lower score (95% CI: −0.75, −0.35). In the adjusted model, none of the differences in sleep health score among the profiles remained statistically significant. Furthermore, the sleep health score was 0.11 points lower for each one-hour increase in the hours between wake time and first eating (95% CI: −0.14, −0.09). The sleep health score was also 0.05 points lower for each one-hour increase in the hours between the last eating and bedtime (95% CI: −0.07, −0.03), while an additional hour in the eating window increased the sleep health score by 0.05 points (95% CI: 0.02, 0.07). However, after adjustment, these associations were attenuated, with only the hours between wake time and first eating associated with sleep health score (95% CI: −0.14, −0.02) (Table 3).

### 3.4. Chrononutrition Profiles and Sleep Health

**Figure 1:**
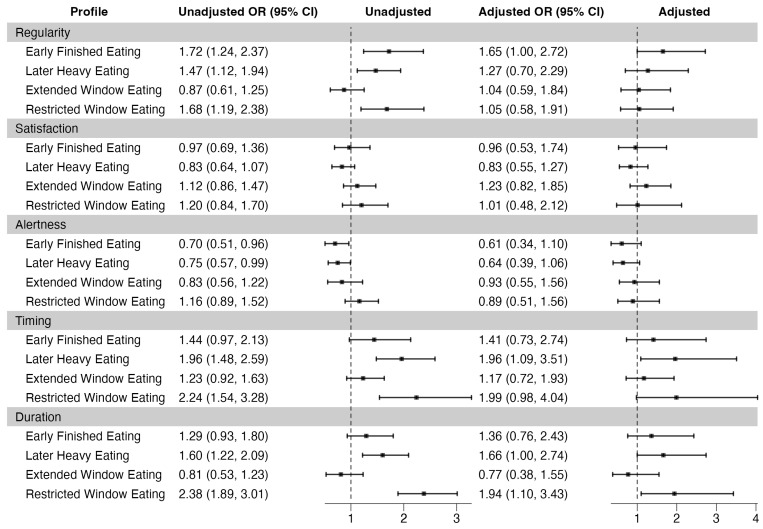
Associations between chrononutrition profiles and sleep health. Reference is Typical Eating Profile. Adjusted for age, race, education, total daily calories, income, marital status, depression, healthy eating index, alcohol use, caffeine, and physical activity.

After adjustment, the Later Heavy Eating profile showed 96% higher odds of poor sleep timing (95% CI: 1.09, 3.51), with a difference of 55 min from 3 a.m. (95% CI: 21.81, 88.08) compared to the Typical Eating profile. The Restricted Window Eating profile had 94% higher odds of poor sleep duration (95% CI: 1.10, 3.43), with a difference of 0.34 h from 8 h duration (95% CI: 0.12, 0.56). The profiles had no significant difference in regularity, alertness, or satisfaction (Figure 1 and Table 4).

However, in our unadjusted model, the Early Finished Eating, the Later Heavy Eating, and the Restricted Window Eating profiles had higher odds of poor regularity (OR = 1.72 95% CI: 1.24, 2.37; OR = 1.47 95% CI: 1.12, 1.94; OR = 1.68 95% CI: 1.19, 2.38, respectively). Additionally, the Early Finished Eating and Later Heavy Eating profiles demonstrated 30% (95% CI: 0.51, 0.96) and 25% (95% CI: 0.57, 0.99) lower odds of poor alertness. The Restricted Window Eating profile showed 124% higher odds of poor sleep timing (95% CI: 1.54, 3.28), and the Later Heavy Eating profile had 60% higher odds of poor sleep duration (95% CI: 1.22, 2.09), but these associations were attenuated after adjustment (Figure 1 and Table 5).

### 3.5. Chrononutrition Behaviors and Sleep Health

There were associations between chrononutrition behaviors and individual sleep health domains. Each one-hour increase in the hours between wake time and first eating was associated with an 19% increase in the odds of poor sleep timing (95% CI: 1.07, 1.33), with a timing difference from 3 a.m. increasing by 21.14 min (95% CI: 10.32, 31.97). Additionally, each one-hour increase in this interval was linked to 21% higher odds of poor sleep duration (95% CI: 1.09, 1.33). Each one-hour increase in the hours between last eating and bedtime was associated with the higher odds of poor sleep duration by 9% (95% CI: 1.03, 1.16). A one-hour increase in the eating window was associated with 10% lower odds of poor sleep timing (95% CI: 0.84, 0.98).

**Figure 2:**
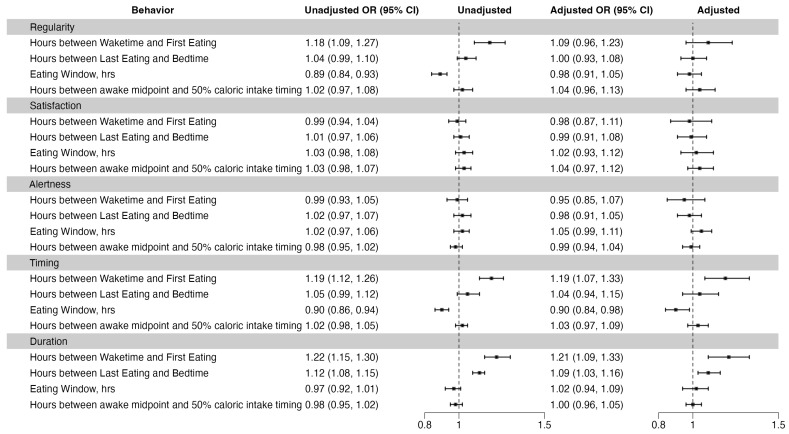
Associations between chrononutrition behaviors and sleep health. Adjusted for age, race, education, total daily calories, income, marital status, depression, healthy eating index, alcohol use, caffeine, and physical activity.

In the unadjusted analysis, each additional hour between wake time and first eating was associated with 18% higher odds of poor regularity (95% CI: 1.09, 1.27). An additional hour in the eating window was associated with 11% lower odds of poor regularity (95% CI: 0.84, 0.93). However, these associations were attenuated after adjustment. The results for the hours between last eating and bedtime were similar before and after adjustment. There was no significant relationship between the hours from the awake midpoint to 50% caloric intake timing and any sleep health domain (Figure 2).

## 4. Discussion

Our study aimed to identify chrononutrition profiles based on individuals’ chrononutrition behaviors and explore the associations between these chrononutrition patterns and multidimensional sleep health in a nationally representative sample of U.S. adults. We identified five distinct chrononutrition profiles: Typical Eating, Early Finished Eating, Later Heavy Eating, Extended Window Eating, and Restricted Window Eating.

The composite sleep health score was lower for the Later Heavy Eating and Restricted Window Eating profiles compared to the Typical Eating profile. However, after adjustment for covariates, the differences in sleep health score were not statistically significant. For chrononutrition behaviors, an increase in the hours between wake time and first eating and the hours between last eating and bedtime was associated with lower multidimensional sleep health scores, whereas an increase in the eating window duration was associated with higher scores. After adjustment, only the increase in the hours between wake time and first eating remained significantly associated with a lower sleep health score.

Compared to the Typical Eating profile, the Later Heavy Eating profile exhibited poorer sleep timing, while the Restricted Window Eating profile showed poorer sleep duration compared to Typical Eating profile. Among chrononutrition behaviors, an increase in the hours between wake time and first eating was associated with poorer sleep timing and duration, while an increase in the hours between last eating and bedtime was linked to poorer sleep duration. An increase in the eating window was associated with better sleep timing. In the unadjusted model, an increase in the hours between wake time and first eating was associated with poorer sleep regularity, and an increase in the eating window was associated with better sleep regularity, which was attenuated in fully adjusted models.

Our study represents the first investigation to characterize chrononutrition patterns and their association with sleep health using a representative sample of U.S. adults. Our findings partially align with and expand upon previous related research. In a study by Faris et al. involving 498 university students, skipping breakfast was associated with poorer sleep quality as measured by the Pittsburgh Sleep Quality Index (PSQI) [21]. Among 1608 university students examined in the study by de-Arruda et al., skipping breakfast and skipping lunch were linked to short sleep duration [50]. Gwin and Leidy, in a randomized crossover-design study with 13 participants aged 20–32, found that consuming breakfast, compared to skipping it, resulted in significantly shorter total sleep duration and marginally higher sleep quality [51]. In our study, an increase in the hours between wake time and first eating was associated with poorer sleep timing and duration (either shorter or longer), but there was no association with sleep quality.

Chung et al. examined the associations between evening meal timing and sleep quality among 793 university students. Eating within 3 h of bedtime was associated with higher odds of nocturnal awakening at least once, but was not linked to poor sleep duration, defined as 7 h or less [22]. In a study by Iao et al. involving USA residents aged 15 years and older, eating 1 h before bedtime was associated with longer sleep than recommended and increased wake after sleep onset [52]. According to Kim et al., eating before breakfast or after bedtime was linked to short sleep duration among women in the NIEHS Sister Study cohort, including those from the USA and Puerto Rico [53]. Yu and Lam, in a study of 215 participants aged 15 to 24 years in Hong Kong, found that nighttime eating was associated with poorer sleep quality per the PSQI [54]. Yasuda et al., in a study of 270 young Japanese individuals aged 18–40 years, divided participants into tertiles based on the time from dinner to bedtime. They found no significant differences in sleep duration or sleep quality according to PSQI among these groups [55]. Teoh et al. identified that eating closer to bedtime was associated with the peak time of melatonin secretion being further misaligned with the middle of the sleep period among women experiencing their first pregnancy in Malaysia [56]. In our study, an increase in the hours between last eating and bedtime was associated with poorer sleep duration but was not related to sleep quality. The differing results regarding sleep quality may be due to our study defining sleep quality based on doctor-reported sleep problems.

Kesztyüs et al. observed that an 8–9 h time-restricted eating window over three months was linked to better sleep quality, rated on a 0–100 scale, but had no association with sleep duration [57]. In a 14-week RCT, Steger et al. reported that an 8 h time-restricted eating window resulted in shorter sleep duration compared to a 12 h window [58]. Simon et al., through a secondary analysis of the See Food Study, identified that a shorter eating window was associated with longer sleep duration among participants with overweight or obesity [59]. Cienfuegos et al.’s RCT examined the effects of 4 h and 6 h eating windows and noted no effect on sleep quality according to the PSQI and sleep duration compared to the usual meal timing group [60]. Previous studies have shown inconsistent associations of sleep duration and quality with eating windows, and our study revealed no significant differences.

The association between chrononutrition patterns and sleep health is intricate and multifaceted, encompassing various physiological systems. Circadian rhythms, the internal biological clocks in organisms, orchestrate various physiological processes across an approximately 24 h cycle, essential for the regulation of daily physiological functions. Hormones such as ghrelin and leptin, which are pivotal in appetite regulation, display circadian variations that are synchronized with the sleep–wake cycle [61]. This hormonal rhythm can influence eating behaviors, linking chrononutrition patterns to sleep patterns. Additionally, dietary patterns can significantly alter circadian rhythms, as the timing of food/beverage consumption acts as a strong zeitgeber (time cue) for the body’s peripheral clocks, thereby impacting sleep schedules [62]. Metabolic processes also play a critical role; different metabolic states can affect the quality and duration of sleep, indicating a deep-seated connection between metabolism and sleep health [63].

While our study contributes significant insights into the association between chrononutrition patterns and sleep health, it has some limitations. Firstly, the cross-sectional design of the study inherently limits our ability to infer temporal relationships between chrononutrition patterns and sleep health outcomes. Future studies with longitudinal designs are needed to better understand the directionality and causality of these relationships. Secondly, the recommended method for dietary assessment includes at least two 24 h dietary recalls, with three being optimal, including one collected on the weekend [64,65]. This study included two recalls per participant, potentially limiting the capture of usual dietary intake. However, our approach was designed to balance accurate assessment and feasibility (e.g., participant burden, research resources) of data collection in a nationally representative study population. Additionally, we calculated the difference between the mean waketime/bedtime and the mean first/last meal timing, rather than calculating daily differences and averaging them, which may have introduced bias by not capturing day-to-day variability. While we included social determinants, such as income and education, that may influence chrononutrition patterns in our regression models, there may be unobserved social determinants that affect both chrononutrition patterns and sleep health. Thus, unmeasured confounding could bias our results towards or away from the null.

An additional limitation is that sleep satisfaction was inferred from whether participants had heard about sleep problems from a doctor, which may not fully capture the breadth of sleep quality. This approach, while practical, may oversimplify the complexity of sleep satisfaction and its relationship with chrononutrition patterns, leading to non-differential misclassification. The lack of consensus on defining good sleep health for certain domains, such as sleep timing and regularity, presented challenges. To navigate this, we based our definitions on the expertise within our research team and insights from prior studies [11,28]. Additionally, we conducted a sensitivity analysis, ensuring the robustness of our findings despite these challenges. Lastly, the reliance on self-reporting for capturing sleep health indicators, while practical for a large-scale study, introduces inherent limitations. While self-reporting is useful for assessing domains like sleepiness and satisfaction, it differs from objective measures in estimating other domains (e.g., sleep duration) [66]. However, participants’ perceptions of their sleep are independently associated with sleep health outcomes [67].

Despite the limitations, this study has strengths that contribute significantly to its robustness and the reliability of its findings. The large-scale nature of the study stands out as a primary strength. Utilizing a representative sample of U.S. adults from the NHANES allowed for comprehensive insights into the chrononutrition patterns of the general population and their associations with sleep health. Furthermore, our innovative approach to characterizing chrononutrition profiles represents a significant advancement in chrono-nutrition and sleep health. This study is the first to utilize NHANES to identify distinct chrononutrition patterns within the U.S. adult population, offering a groundbreaking perspective on the relationship between chrononutrition patterns and sleep health.

In conclusion, this descriptive study marks a significant stride in elucidating the association between chrononutrition patterns and sleep health. Consuming a higher proportion of calories later in the day was associated with poorer sleep timing, and those who had shorter eating windows also experienced poorer sleep duration. These findings advocate for integrating chrononutrition patterns into dietary guidelines and emphasize the potential of tailored dietary interventions to enhance sleep health. As a first step in characterizing these patterns in a representative U.S. adult population, this research provides a foundation that generates hypotheses for future research and could lead to evidence-based public health recommendations. Ultimately, this study contributes to a more comprehensive understanding of how chrononutrition patterns influence sleep health, aiming to inform public health strategies and dietary guidelines that enhance sleep health across the population, thereby fostering improved overall well-being.

## 5. Conclusions

Our findings highlight the potential for developing personalized chrononutrition interventions to improve sleep health in future research.

## Figures and Tables

**Table 1:** Demographic and behavioral characteristics.

	Chrononutrition Profiles					
Characteristic	Typical Eating, N = 58,724,157	Early Finished Eating, N = 38,579,236	Later Heavy Eating, N = 37,694,415	Extended Window Eating, N = 35,857,662	Restricted Window Eating, N = 19,084,566	*p*-Value ^3^
Sex						0.93
Male	31%	20%	20%	19%	11%	
Female	31%	20%	20%	19%	9.5%	
Age, years ^1^	49 (35, 65)	43 (28, 59)	47 (33, 61)	55 (39, 66)	41 (26, 58)	<0.001
Race/ethnicity						<0.001
Mexican American	26%	20%	26%	15%	13%	
Other Hispanic	24%	23%	22%	17%	14%	
Non-Hispanic White	34%	20%	17%	21%	6.7%	
Non-Hispanic Black	19%	17%	30%	11%	24%	
Other Race—Including Multi-Racial	32%	21%	19%	17%	12%	
Education						<0.001
Below High School	24%	21%	24%	14%	17%	
High School	29%	23%	20%	15%	13%	
Beyond High School	34%	19%	19%	21%	7.1%	
Sedentary Time, min/d ^1^	300 (240, 480)	360 (240, 480)	300 (180, 480)	300 (240, 480)	300 (180, 480)	0.23
Body mass index, kg/m^2 2^	29 (6)	29 (7)	30 (7)	29 (6)	30 (8)	0.34
Body mass index						0.035
Underweight	28%	37%	14%	3.4%	18%	
Normal	31%	20%	20%	19%	10%	
Overweight	31%	21%	17%	22%	8.5%	
Obesity	31%	19%	22%	17%	11%	
Total kcal ^2^	2037 (705)	1868 (741)	2107 (800)	2230 (829)	1872 (887)	<0.001
Working Status						0.30
Full Time	29%	21%	22%	19%	9.0%	
Not Working	33%	20%	17%	19%	11%	
Part Time	29%	21%	20%	20%	11%	
Work Schedule						<0.001
Traditional 9 a.m.–5 p.m.	29%	21%	23%	22%	5.4%	
Evening or nights	16%	33%	15%	8.3%	27%	
Early mornings	20%	17%	31%	19%	13%	
Variable	35%	19%	19%	18%	9.9%	
Income level ^4^						<0.001
Above 1.85	35%	20%	18%	20%	7.3%	
Between 1.30 and 1.85	25%	19%	22%	19%	15%	
Below 1.30	23%	24%	22%	14%	18%	
Partner						<0.001
Married/Partner	34%	19%	19%	21%	7.3%	
Single	26%	22%	22%	16%	14%	
Current Smoking	30%	21%	16%	19%	14%	0.042
Depression	29%	19%	15%	17%	21%	0.031
Alcohol use						0.24
Heavy	31%	21%	20%	17%	10%	
Moderate	34%	18%	19%	20%	8.7%	
No	23%	23%	23%	19%	12%	
Health Eating Index ^2^	53 (12)	50 (13)	50 (12)	53 (12)	45 (12)	<0.001
On Special Diet	34%	18%	19%	19%	9.1%	0.57
Caffeine (mg) ^1^	151 (61, 252)	99 (31, 195)	104 (34, 190)	164 (84, 288)	69 (14, 144)	<0.001
Carbohydrate Percentage ^2^	45 (10)	45 (10)	46 (9)	46 (9)	47 (11)	0.47
Physical activity, >150 min/week	31%	21%	18%	20%	9.3%	0.019
Supplement Use	31%	21%	19%	24%	4.2%	0.67

^1^ Median (IQR). ^2^ Mean (SD). We reported weight frequencies, i.e., the number of people each subject represents in the US adult population. ^3^ Chi-squared test with Rao and Scott’s second-order correction; Wilcoxon rank-sum test for complex survey samples. ^4^ Income was assessed through the family monthly poverty level index according to the HHS poverty guidelines [49].

**Table 2:** Chrononutrition behaviors by profiles.

	Chrononutrition Profile					
Characteristic	Overall, N = 189,940,036 ^1^	Typical Eating, N = 58,724,157 ^1^	Early Finished Eating, N = 38,579,236 ^1^	Later Heavy Eating, N = 37,694,415 ^1^	Extended Window Eating, N = 35,857,662 ^1^	Restricted Window Eating, N = 19,084,566 ^1^
Hours between Waketime and First Eating	1.63 (1.42)	0.83 (0.52)	1.54 (0.88)	2.83 (0.85)	0.81 (0.58)	3.44 (2.50)
Hours between Last Eating and Bedtime	2.83 (1.97)	2.67 (0.74)	4.22 (0.88)	1.69 (0.84)	1.01 (0.54)	6.16 (3.07)
Eating Window, hours	12.04 (2.09)	12.74 (0.87)	10.50 (0.99)	11.80 (1.19)	14.39 (0.93)	9.01 (2.95)
Hours between awake midpoint and 50% caloric intake timing	0.53 (2.57)	0.11 (2.38)	−0.50 (2.41)	1.68 (2.29)	1.27 (2.49)	0.27 (2.91)

^1^ Mean (SD). We reported weight frequencies, i.e., the number of people each subject represents in the US adult population.

**Table 3:** Associations between chrononutrition patterns and composite sleep health score.

	Unadjusted		Adjusted	
Characteristic	Beta	95% CI ^1^	Beta	95% CI ^1^
Hours between Waketime and First Eating	**−0.11**	**−0.14**, **−0.09**	**−0.08**	**−0.14**, **−0.02**
Hours between Last Eating and Bedtime	**−0.05**	**−0.07**, **−0.03**	−0.02	−0.06, 0.01
Eating Window, h	**0.05**	**0.02**, **0.07**	0.01	−0.03, 0.05
Hours between awake midpoint and 50% caloric intake timing	−0.01	−0.03, 0.01	−0.02	−0.05, 0.01
Chrononutrition profile (reference: Typical Eating)				
Early Finished Eating	−0.16	−0.32, 0	−0.13	−0.43, 0.17
Later Heavy Eating	**−0.23**	**−0.37**, **−0.1**	−0.18	−0.45, 0.09
Extended Window Eating	0.03	−0.15, 0.2	−0.02	−0.29, 0.25
Restricted Window Eating	**−0.55**	**−0.75**, **−0.35**	−0.29	−0.69, 0.12

^1^ CI = confidence interval. Significant results are bolded. Adjusted for age, race, education, total daily calories, income, marital status, depression, healthy eating index, alcohol use, caffeine, and physical activity.

**Table 4:** Associations between chrononutrition patterns and sleep health (adjusted model).

	Midpoint Standard Deviation (min)		Difference in Timing (min)		Difference in Duration (h)	
Characteristic	Beta	95% CI	Beta	95% CI	Beta	95% CI
Hours between Waketime and First Eating	−6.51	−31.3, 18.29	**21.14**	**10.32**, **31.97**	**0.10**	**0.06**, **0.14**
Hours between Last Eating and Bedtime	4.70	−10.58, 19.98	−0.19	−4.32, 3.94	**0.04**	**0.01**, **0.07**
Eating Window, hours	−1.78	−15.02, 11.46	**−6.74**	**−11.14**, **−2.35**	0.00	−0.03, 0.03
Hours between awake midpoint and 50% caloric intake timing	−1.12	−13.07, 10.82	2.22	−0.84, 5.28	0.00	−0.02, 0.02
Chrononutrition profile (reference: Typical Eating)						
Early Finished Eating	**121.47**	**22.05**, **220.88**	**18.80**	**8.86**, **28.74**	0.18	−0.01, 0.36
Later Heavy Eating	−25.08	−146.18, 96.02	**54.94**	**21.81**, **88.08**	**0.20**	**0.01**, **0.39**
Extended Window Eating	9.72	−96.12, 115.56	12.18	−2.63, 26.98	−0.06	−0.28, 0.17
Restricted Window Eating	−24.30	−111.67, 63.07	**66.76**	**18.52**, **114.99**	**0.34**	**0.12**, **0.56**

Significant results are bolded. Adjusted for age, race, education, total daily calories, income, marital status, depression, healthy eating index, alcohol use, caffeine, and physical activity.

**Table 5:** Associations between chrononutrition patterns and sleep health (unadjusted model).

	Midpoint Standard Deviation (min)		Difference in Timing (min)		Difference in Duration (h)	
Characteristic	Beta	95% CI	Beta	95% CI	Beta	95% CI
Hours between Waketime and First Eating	12.27	−4.65, 29.18	**21.37**	**12.25**, **30.49**	**0.11**	**0.08**, **0.13**
Hours between Last Eating and Bedtime	**11.84**	**2.18**, **21.5**	−0.87	−4.28, 2.53	**0.05**	**0.04**, **0.07**
Eating Window, hours	**−17.67**	**−27.31**, **−8.02**	**−5.80**	**−9.18**, **−2.43**	−0.02	−0.03, 0
Hours between awake midpoint and 50% caloric intake timing	0.16	−9.31, 9.63	**3.36**	**0.63**, **6.09**	−0.01	−0.02, 0.01
Chrononutrition profile (reference: Typical Eating)						
Early Finished Eating	**125.15**	**54.68**, **195.63**	**17.32**	**6.44**, **28.2**	**0.18**	**0.07**, **0.29**
Later Heavy Eating	15.16	−47.63, 77.94	**61.92**	**40.99**, **82.85**	**0.19**	**0.09**, **0.28**
Extended Window Eating	−11.95	−82.06, 58.15	**12.71**	**0.05**, **25.37**	−0.05	−0.17, 0.07
Restricted Window Eating	53.99	−15.43, 123.41	**62.17**	**29.9**, **94.45**	**0.50**	**0.35**, **0.66**

Significant results are bolded.

## Data Availability

The original data presented in the study are openly available in the NHANES.

## Supplementary Materials

The following supporting information can be downloaded at: https://www.mdpi.com/article/10.3390/nu16213724/s1, Figure S1: Study Population Flowchart; Figure S2: Determination of Optimal Latent Classes Using AIC/BIC; Table S1: Descriptive characteristics by including status in analysis.
