# Supplementary material for: Association Between Chrononutrition Patterns and Multidimensional Sleep Health
Source: Nutrients. 2024 Oct 31;16(21):3724. doi: 10.3390/nu16213724 (PMC11547175; doi:10.3390/nu16213724)

Supplemental Table S1. Descriptive characteristics by including status in analysis

Characteristic	Overall N = 247,947,060[1]	Included in Analysis: No N = 105,429,704[1]	Included in Analysis: Yes N = 142,517,356[1]	p-value[2]
Sex				0.61
Male	48%	49%	48%	
Female	52%	51%	52%	
Age, years	47 (32, 61)	44 (30, 60)	49 (34, 63)	<0.001
Race/ethnicity				<0.001
Mexican American	8.6%	9.2%	8.1%	
Other Hispanic	7.7%	8.9%	6.9%	
Non-Hispanic White	62%	58%	65%	
Non-Hispanic Black	11%	12%	11%	
Other Race - Including Multi-Racial	10%	11%	9.2%	
Education				<0.001
Below High School	11%	14%	8.7%	
Beyond High School	62%	57%	66%	
High School	27%	29%	26%	
Sedentary Time, min/d	300 (180, 480)	300 (180, 480)	300 (240, 480)	0.044
Body mass index, kg/m2	29 (25, 34)	29 (25, 34)	29 (25, 33)	0.72
Working Status				0.79
Full Time	47%	48%	47%	
Not Working	37%	37%	37%	
Part Time	16%	15%	16%	
Work Schedule				<0.001

Characteristic	Included in Analysis			p-value[2]
	**Overall**<br>N = 247,947,060[1]	**No**<br>N = 105,429,704[1]	**Yes**<br>N = 142,517,356[1]	
Traditional 9am-5pm	41%	34%	46%	
Evening or nights	10%	17%	5.2%	
Early mornings	13%	12%	13%	
Variable	36%	37%	36%	
Income level				<0.001
Above 1.85	70%	65%	73%	
Below 1.30	19%	23%	17%	
Between 1.30 and 1.85	11%	12%	9.8%	
Partner				0.008
Married/Partner	62%	59%	64%	
Single	38%	41%	36%	
Smoking status				0.002
Current Smoking	23%	27%	20%	
Non-Smoking	77%	73%	80%	
Depression				0.15
Depression	3.2%	3.6%	3.0%	
No Depression	97%	96%	97%	
Alcohol use				0.005
Heavy	45%	50%	43%	
Moderate	46%	41%	48%	
No	9.1%	9.7%	8.8%	
Physical activity, >150 min/wk	65%	64%	66%	0.30

[1]%; Median (Q1, Q3)

[2]Pearson's X^2: Rao & Scott adjustment; Design-based KruskalWallis test

Figure S1: Study Population Flowchart;

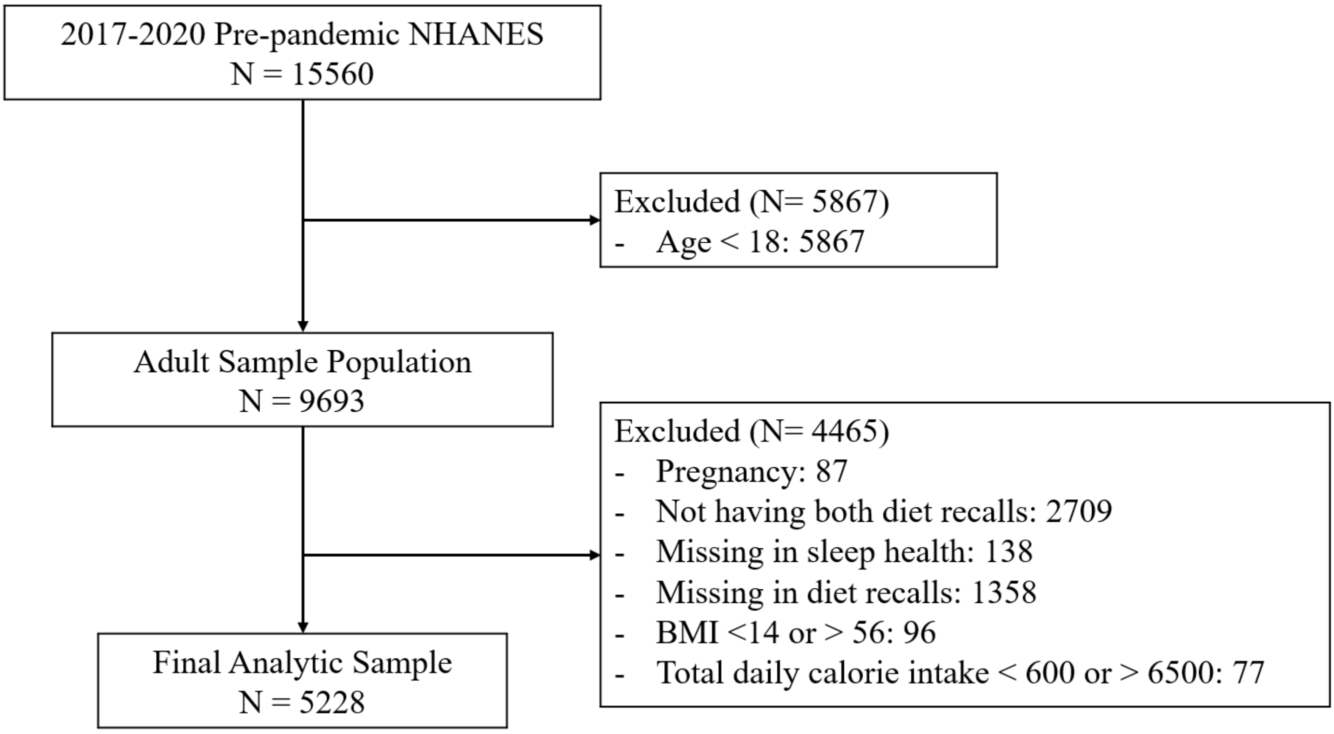

Figure S2: Determination of Optimal Latent Classes Using AIC/BIC

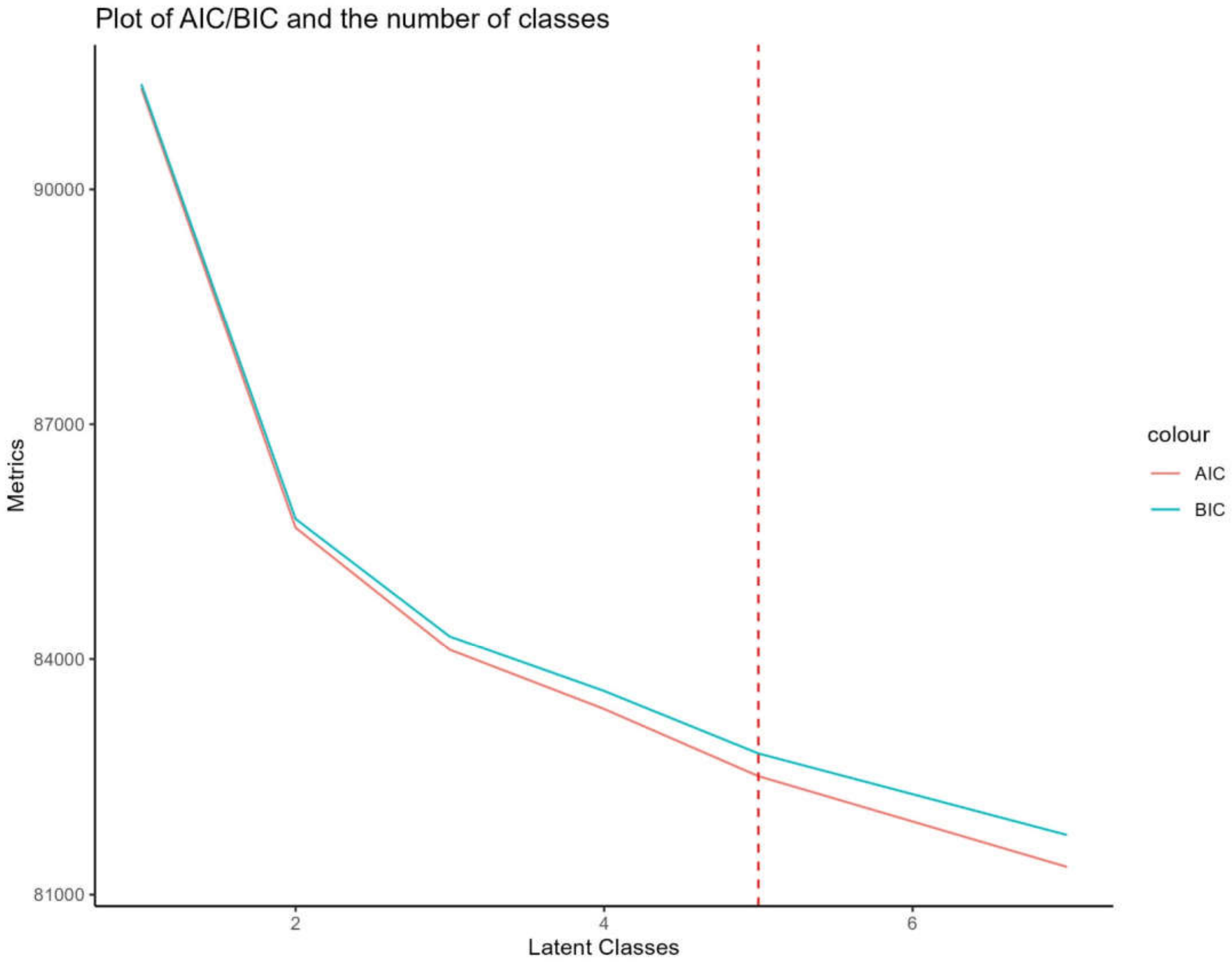

Supplement: Supplementary file 1 [file nutrients-16-03724-s001.zip › nutrients-3213866-supplementary.pdf]
